# Supplementary material for: Coherence mapping to identify the intermediates of multi-channel dissociative ionization
Source: Commun Chem. 2024 May 9;7:103. doi: 10.1038/s42004-024-01176-5 (PMC11549452; doi:10.1038/s42004-024-01176-5)
Supplement: Supplementary file 2 — Supplemental material [file 42004_2024_1176_MOESM2_ESM.pdf]

Coherence Mapping to Identify the Intermediates of Multi-Channel Dissociative Ionization

Jacob Stamm[1], Sung Kwon[1], Shawn Sandhu[1], Jesse Sandhu[1], Benjamin G. Levine[2,3], Marcos Dantus[1,4*]

[1]Department of Chemistry, Michigan State University, S Shaw Ln, East Lansing, 48824, Michigan, USA.
[2]Department of Chemistry, Stony Brook University, John S. Toll Drive, Stony Brook, 11794, New York, USA.
[3]Institute for Advanced Computational Science, Stony Brook University, IACS Building,, Stony Brook, 11794, New York, USA.
[4]Department of Physics and Astronomy, Michigan State University, Wilson Rd, East Lansing, 48824, Michigan, USA.

*Corresponding author(s). E-mail(s): dantus@msu.edu;

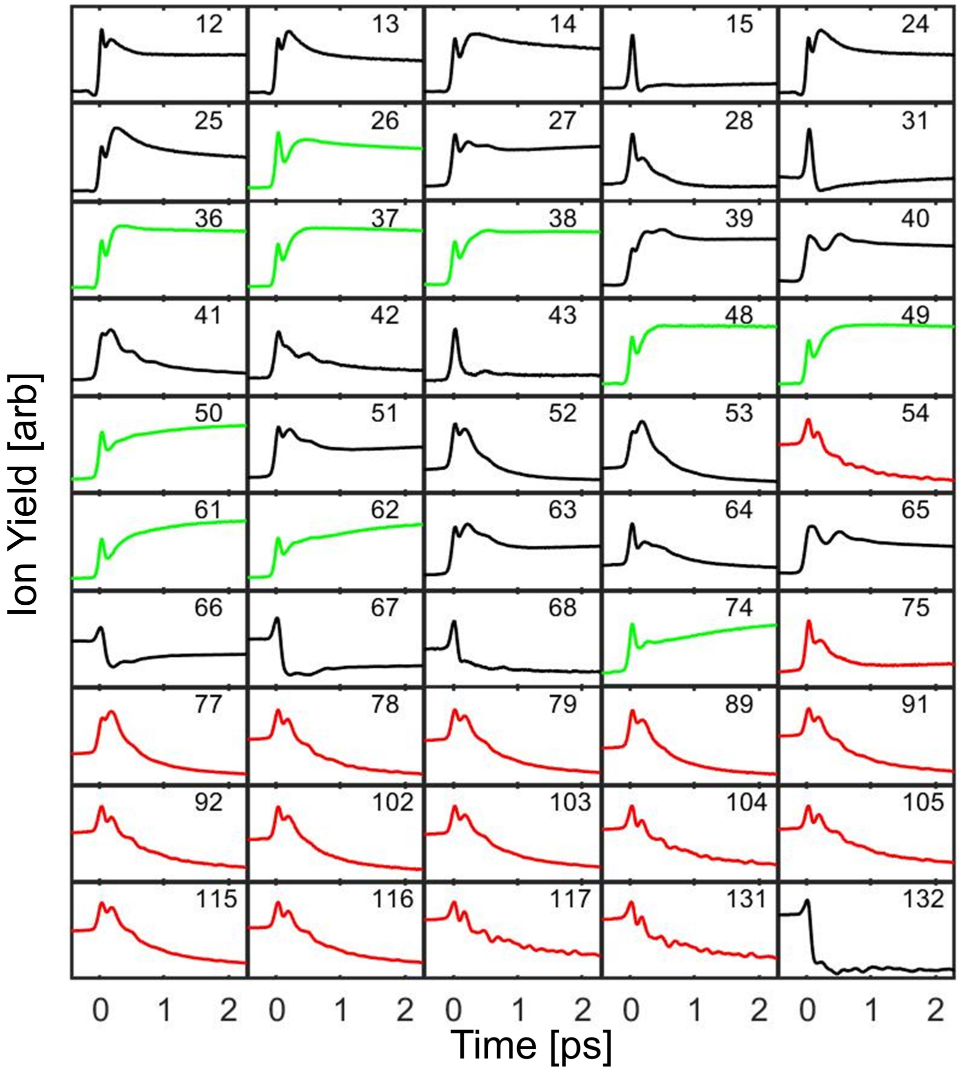

Supplementary Figure 1: Time-resolved ion yields for the 50 highest yield ions. Some transients are colored red or green to indicate similarities. Green: Ions showing a general enhancement following time zero with a brief drop in yield and a subsequent recovery. Red: Ions shows a long depletion following time zero. These red transients comprise almost all ions that show the high frequency (135 cm^{-1} and 195 cm^{-1}) modes.

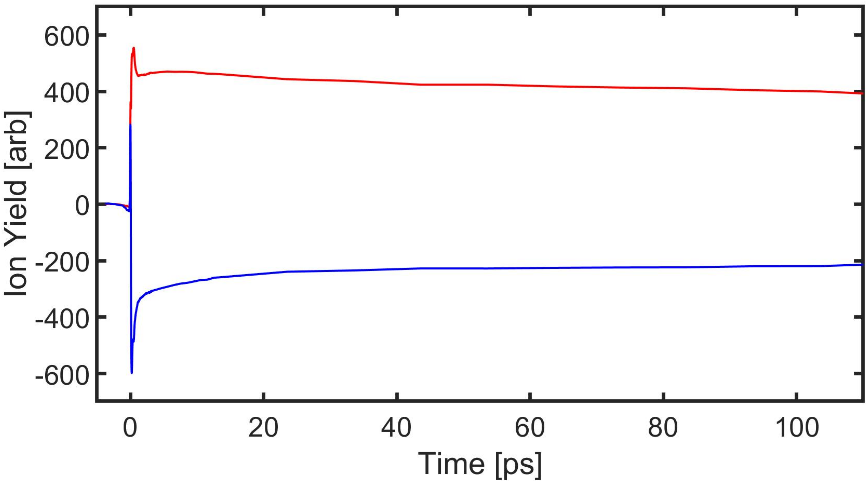

Supplementary Figure 2: Time-resolved ion yields for m/z 39 (red) and m/z 66 (blue). Unlike the time-resolved curves shown elsewhere, these curves show raw ion yields normalized by subtraction so that the yield at negative times is 0. The fact that the curve for m/z 39 is complementary to m/z 66 at long time delays indicates that the probe is fragmenting m/z 66 into m/z 39.

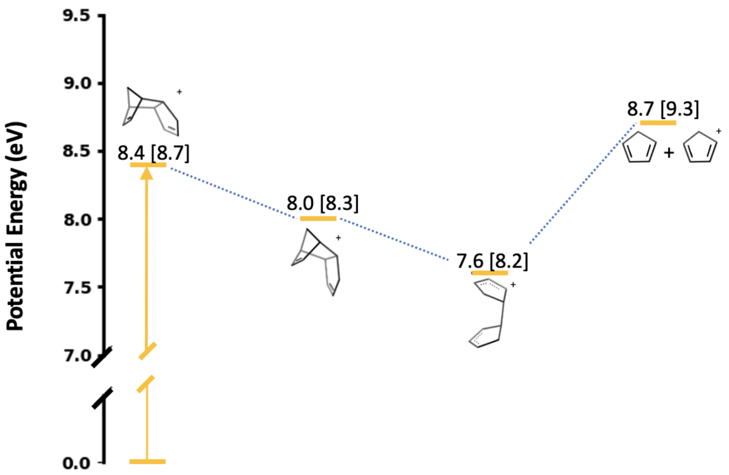

Supplementary Figure 3: Reaction pathway for the formation of cyclopentadiene and a singly charged cyclopentadiene (CPD) from endo-dicyclopentadiene (DCPD). The minimized geometries were optimized using uB3LYP/6-31++G**. The energies along the pathway were obtained using B3LYP and CR-CC(2,3). The CR-CC(2,3) energies are given in brackets. All energies are reported relative to the neutral endo-dicyclopentadiene ground state. We found excellent agreement in the relative adiabatic relaxation energies between uB3LYP and CR-CC(2,3). Despite this reaction being energetically uphill, it can be observed because entropy strongly disfavors the reverse reaction.
