# Supplementary material for: Coherence mapping to identify the intermediates of multi-channel dissociative ionization
Source: Commun Chem. 2024 May 9;7:103. doi: 10.1038/s42004-024-01176-5 (PMC11549452; doi:10.1038/s42004-024-01176-5)
Supplement: Supplementary file 3 — Description of Additional Supplementary Files [file 42004_2024_1176_MOESM3_ESM.pdf]

Description of Additional Supplementary Files

File name: Supplementary Data 1

Description: Ground state structure coordinates; Intact cation structure coordinates; Cation with single bond broken structure; neutral product; product cation; sample TeraChem input

File name: Supplementary Movie 1

Description: AIMD trajectory showing the stepwise mechanism of the rDA reaction of singly-ionized DCPD.

File name: Supplementary Movie 2

Description: AIMD trajectory showing the concerted mechanism of the rDA reaction of singly-ionized DCPD.
